# Activity Patterns in Latissimus Dorsi and Sternocleidomastoid in Classical Singers

**DOI:** 10.1016/j.jvoice.2011.04.008

**Published:** 2012-05

**Authors:** Alan H.D. Watson, Caitlin Williams, Buddug V. James

**Affiliations:** ∗ School of Biosciences, Cardiff University, Cardiff, Wales, UK; † Department of Vocal and Opera Studies, Royal Welsh College of Music and Drama, Cardiff, Wales, UK

**Keywords:** Respiration, Voice, Coloratura, Vibrato, Vocal projection, Posture, Electromyography, Plethysmography

## Abstract

**Objectives:**

The aim of this study was to investigate and compare the roles of the accessory respiratory muscles, latissimus dorsi (LD), and sternocleidomastoid, in classical singing.

**Methods:**

Electromyography was used to record the activity of these muscles in six classically trained female singers carrying out a number of singing and nonsinging tasks. Movements of the chest and abdominal walls were monitored simultaneously using inductive plethysmography, and the sound of the phonations was recorded.

**Results:**

In normal breathing, LD is active transiently during very deep inhalations and in inhalation against resistance. During exhalation it becomes active again as residual capacity is approached or when air is expelled with great force. Sternocleidomastoid (SCM) supports inhalation when lung volume nears 100% vital capacity or when this is very rapid. All singers engaged LD in supported singing where it was associated with maintaining an expanded thorax. In coloratura singing, pulses of activity of increasing amplitude were often seen in LD toward the end of the breath. These were synchronized with each note. During a short phrase typical of the end of an aria, which was sung at full volume with the projected voice, both LD and SCM were active simultaneously. Spectral analysis of muscle activity demonstrated that in some singers, activity in LD and more rarely SCM, fluctuated in phase with vibrato.

**Conclusions:**

LD appears to play a significant role in maintaining chest expansion and the dynamic processes underlying vibrato and coloratura singing in classically trained singers.

## Introduction

An understanding of respiratory muscle activity is central to singing pedagogy, but until recently this has not been based on objective evidence. Although this is beginning to change,^1,2^ there is still much to be learned, and considerable controversy persists over the role of the so-called accessory muscles of respiration.^3–5^ Nowhere is this more true than for latissimus dorsi (LD). Although often referred to in manuals on singing technique, it may be vaguely described as a postural muscle of the back,^6,7^ being active during expiration as one of the “outer dorsal muscles”^8^ or be dismissed as associated with clavicular breathing,^3^ which is discouraged in good vocal technique.

LD is a large flat muscle that lies superficially on the back and takes its origin from the spines of the lower six thoracic vertebrae and, *via* the thoracolumbar fascia, from the lumbosacral vertebrae and the posterior iliac crest.^9^ It passes over the inferior angle of the scapula (to which it is often attached) and inserts into the intratubercular groove of the humerus. Its main action is to adduct, extend, and medially rotate the humerus. As a result, it is particularly well developed in swimmers who must pull the arm downwards against resistance to propel the body through the water. Its role as an accessory muscle of respiration is a consequence of its attachment to the lateral aspect of the lowest 3–4 ribs.^9,10^ The muscle slips responsible are generally said to arise from the ribs ^11^ and their contraction against the fixed humerus is said to elevate the ribs during deep inspiration.^9,12,13^ Paradoxically, the muscle has also been shown to be active during forced expiration, such as coughing. Under these circumstances, contraction of the muscle as a whole is said to lead to compression of the lower thorax.^12^ How the same attachments to the ribs bring about these two opposite actions (both of which can easily be demonstrated) is usually not made clear.

Most of the studies of the action of LD in breathing have focussed on the possibility of its recruitment in patients experiencing respiratory difficulty, either because of chronic obstructive pulmonary disease or paralysis of the major muscles of respiration after spinal lesions. Activity in LD is seen during inspiration in patients with asthma and emphysema,^14^ in whom the muscle fibers often exhibit hypertrophy.^15^ In normal subjects, its recruitment rises progressively with increasing inspiratory loading (inhaling against resistance).^16,17^

In the context of singing, LD has generally been considered as a possible contributor to inspiration.^3,4^ Although its activity has not been recorded during singing, it has been studied during speech^18,19^ where it is found to be active only toward the very end of expiration. Once lung volume has declined to the level at which elastic recoil can no longer generate sufficient pressure to maintain phonation at the required volume and pitch, there is a successive recruitment of muscles of expiration; first the internal intercostals, then the external oblique, and other muscles of the lateral abdominal wall and next rectus abdominus. Finally, at a volume of about 1.3 L below “midrespiration,” LD becomes active.^18,19^ “Mid-respiration” in these studies appears to be about 150 mLs above functional residual capacity. The possibility that LD may play a related role in singing has not yet been examined.

The primary objective of the present investigation was to examine the role of LD in classically trained singers using electromyography, inductive plethysmography, and audio analysis. By these means, we determined when the muscle was active and how this related to chest or abdominal expansion. It was also possible to relate patterns of activity to features of the sound produced such as the production of individual notes or vibrato. Activity in SCM was also monitored as it is known to be active on deep inspiration, although the desirability of this in singers has again been questioned by some pedagogues as it too has been suggested to be associated with clavicular breathing.^3,4^ Recent studies of singers have however, provided a better appreciation of its probable role.^1^

## Methods

### Subjects

The subjects were six classically trained female singers; three mezzo sopranos, and three sopranos. Mezzo 1 (aged 53 years) has had a long and successful career singing operatic roles in the UK and abroad, whereas mezzos 2 and 3 (both aged 23 years) are advanced postgraduate singing students at the Royal Welsh College of Music and Drama (RWCMD). Soprano 1 (aged 31 years) is well established having had a significant number professional engagements and recently spent a year in advanced training at the Cardiff International Academy of Voice. Soprano 2 (aged 21 years) and soprano 3 (aged 22 years) are undergraduate vocal students at RWCMD. Mezzos 2 and 3 and sopranos 1 and 3 either have been, or are currently pupils of mezzo 1 and would, therefore, be expected to show similarities in technique. All experiments were carried out with informed consent and according to the Helsinki declaration, and were approved under the local ethical procedures of the School of Biosciences, Cardiff University.

### Electromyography

Muscle activity was recorded from LD and SCM using a Powerlab 26 T two-channel system (ADinstruments, Chalgrove, UK) or a CED 1902 amplifier connected to a CED 1401 A/D converter (Cambridge Electronic Design, Cambridge, UK). After skin preparation, pairs of bipolar silver/silver chloride electrodes (Kendall Medi-Trace 100, Tyco Healthcare Group, Mansfield, USA) were attached approximately 2 cm apart on the skin. The innervation zone of SCM lies approximately in the middle of the muscle,^20^ so the electrodes were placed on its lower third. LD does not have a well-defined zone of innervation (see Discussion Section). Its neuromuscular junctions are scattered across all regions of the muscle, and some fibers are even thought to be multiply innervated.^11^ The electrodes were placed over the muscle where it forms the posterior wall of the axilla. This is easy to access and does not lie over other muscles that might contaminate the signal. The ground electrode was placed on the back of the neck at C7. In addition to monitoring the electromyograph (EMG) activity during singing, the signal during maximum contraction of each muscle was also recorded. The EMG signals were sampled at 1 kHz and displayed on a computer using *Labchart 6* (ADinstruments, Chalgrove, UK) or *Spike6* software (Cambridge Electronic Design, Cambridge, UK), which allowed further conditioning and analysis of the data. A high band pass filter (set at either 50 or 90 Hz) was applied to the recorded digital signal to eliminate movement artefacts and reduce cardiac signals that where present. For quantification and further analysis, the absolute value of the EMG trace was integrated with a time constant decay of 50 milliseconds. Sound was also recorded using a Shure C606 microphone (Shure, Waltham Abbey, UK) situated 1 m from the singer, and the signal was passed through an SP-24B preamplifier (Maplin, Rotherham, UK) and then to the CED 1401. The *Spike6* software allowed it to be displayed as a sonogram if required. To determine whether muscle activity was synchronized with auditory events, power analyses were carried out on selected sections of this integrated EMG signal using fast Fourier transform.

### Inductive plethysmography

Inductive plethysmography was carried out using an Inductotrace system (model 10.9000, Ambulatory Monitoring, Arosley, NY, USA). Two inductive respibands were used, one placed around the chest at the level of the axilla and the other around the abdomen, between the lowest rib and iliac crest. The analog signal, which was a function of circumference, was digitized using a CED 1401 A/D converter and displayed with *Spike6* software.

### Experimental breathing tasks

Each singer was asked to carry out the following tasks. They were instructed to keep their arms relaxed and hanging vertically by their sides as they did so.

**Figure 1:**
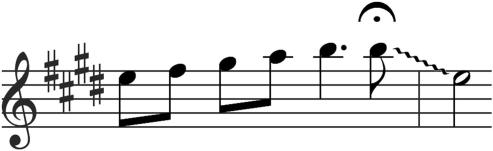
The “aria finale” exercise, taken from “Un voce poco fa” by Rossini.

1. Tidal breathing, then maximal inhalation after which the lungs were then allowed to empty by elastic recoil. Further maximal inhalations followed by slow forced expirations until only the residual volume remained, were then carried out. These and subsequent tasks were performed several times.
2. A series of short (<0.5 second duration) forceful expirations.
3. Resistance breathing, carried out through an Ultrabreathe respiratory trainer (Tangent Healthcare Inc., Peterborough, UK). This provides resistance both to inhalation and exhalation,^21^ and the object of the task was to enable comparisons with previous studies of the activity of LD under respiratory loading.^16,17^
4. After a maximal inhalation, a nonsung vocalization of “ah” was maintained at mezzo piano for as long as possible.
5. A series of notes (do-re-mi-fa-so-fa-mi-re-do) in a comfortable range for the projected voice at mezzo forte. The starting note for the singers lay between C^♯^4 and F^♯^4. This was repeated at least three times with an inhalation in between. Singers were asked to carry out the task first when consciously engaging LD (or chest driven), and then for a second time, consciously without using LD (abdominally driven).
6. A coloratura exercise. A major scale of one octave plus a tone (a 9 note scale), ascending and descending, was sung continuously for each of the five vowels ending with a final arpeggio. This was performed mezzo forte and if possible, under a single breath. It was a challenging exercise and not all singers managed to complete it, but as the object was to see the effect on muscle activity as the amount of air in the lungs declined toward residual volume, noncompletion did not invalidate the object of the exercise. All of the singers started on C4 or C^#^4.
7. Aria finale (Figure 1). This was selected to be typical of the end of an aria (“Una voce poco fa” by Rossini) and was sung at full power with the projected voice. All of the singers started on E5 or E^♭^5.

## Results

### Nonsinging tasks

In the following account, the peak level of muscle activity during particular tasks is expressed as a percentage of the level of activity recorded from the subjects when they were asked to produce a maximal muscle contraction (mean ± standard deviation from the six singers) as measured from the integrated EMG signals. The object of the nonsung tasks was to allow a comparison of activity in LD in our subjects, with previous published studies.

**Figure 2:**
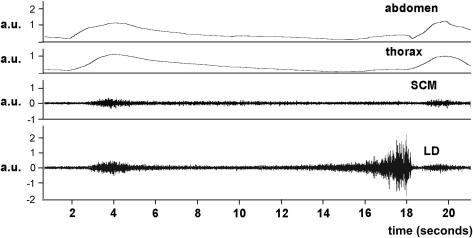
Prolonged breath (task 1). Upper traces show inductive plethysmograph recordings from abdomen and thorax, whereas the lower traces are raw EMG recordings from SCM and LD during a maximal exhalation without phonation (soprano 1). The scale for all traces is in arbitrary units. The same scale is used for the two upper and the two lower traces. a.u., Arbitrary units.

Taking a deep breath and exhaling for as long as possible (task 1) shows that LD is active at two different phases (Figure 2); first, during the initial inhalation that ends close to 100% vital capacity. Muscle activity peaked at 8 ± 6% before falling back within the first few seconds of the exhalation, to levels indistinguishable from background. Toward the end of the exhalation, activity rose progressively to a maximum of 26 ± 13% before ceasing abruptly at the end of the breath. The first phase of activity coincides with an increase in circumference of the chest and abdomen (as shown by inductive plethysmography), but the second phase toward the end of the exhalation does not. A burst of activity is also seen in SCM during the initial strong inhalation (13 ± 11%), but this rapidly falls to a level, which alters little during the rest of the exhalation. The pattern of activity in LD and SCM is essentially similar during the production of an extended nonsung vowel sound (not shown) although the level of activity in LD at the end of the breath does not rise so high (19 ± 12%), presumably because phonation cannot be sustained at the ultimate levels of chest collapse that are reached in its absence.

During a series of sharp exhalations, peaks of activity in LD (23 ± 17%) coincided with abrupt reductions in abdominal circumference (not shown). In some singers chest circumference fell simultaneously, but in others the force of abdominal contraction was sufficient to cause a brief paradoxical increase in chest circumference. Depending on how the exhalation was carried out, activity in SCM was sometimes absent, but where present, it either appeared synchronously with or slightly before activity in LD.

**Figure 3:**
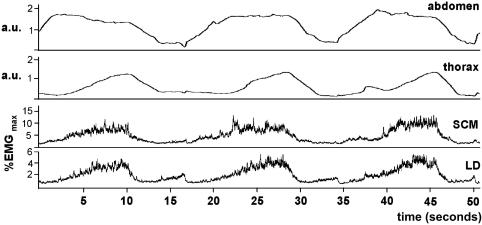
Breathing against resistance (task 3—mezzo 2). Upper traces show inductive plethysmograph recordings and the lower traces show integrated EMG recordings from SCM and LD. The EMG traces as expressed as a percentage of the maximum activity recorded from the muscles. The pattern of EMG activity parallels the changes in thoracic circumference. The same labeling conventions are use in the following figures. a.u., Arbitrary units.

When breathing against resistance (Figure 3), activity in LD tended to rise in phase with increasing chest circumference, peaking at a mean level of 16 ± 3%. Activity in SCM generally followed a similar pattern (20 ± 5%). A small peak in the activity of LD was sometimes apparent right at the end of the exhalation phase.

### Singing tasks

#### Conscious engagement of LD in the projected voice (task 5)

**Figure 4:**
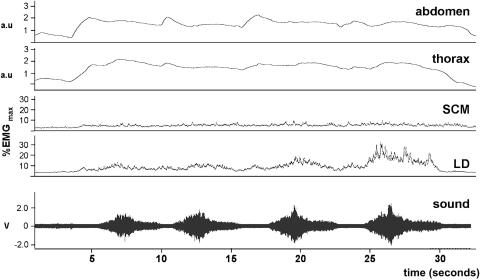
Singing task 5 (soprano 1) with conscious engagement of LD. The loudness of the voice increases with each repetition of the exercise, and this is correlated with a progressive increase in activity in LD. a.u., Arbitrary units.

**Figure 5:**
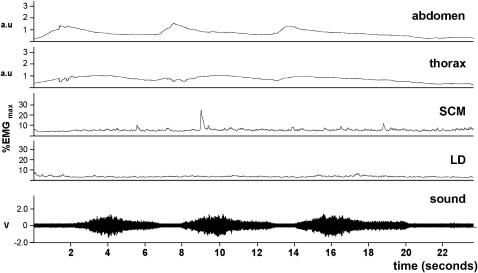
Singing task 5 (soprano 1) with conscious disengagement of LD. There is almost no activity in either LD or SCM and thoracic expansion is greatly reduced compared to Figure 4. a.u., Arbitrary units.

When singers used their trained projected voice, LD was active during the sung phrases (Figure 4). Activity tended to rise and fall with pitch and intensity, peaking at a mean of 12 ± 6%. In Figure 4, the loudness of the voice increased with each repetition of the exercise and level of activity in the muscle rose in parallel. The singers were able on demand to sing without using the muscle (Figure 5). Under these circumstances its peak activity was only 3 ± 1%, and the increase in chest diameter was markedly reduced although there was sometimes no change in the plethysmograph trace for the abdomen. This often led to a reduction in sound intensity. Fourier analysis (not shown) of a sonogram derived from the audio signal from one of the mezzos did not reveal any significant differences in the prominence of the singer’s formant when LD was inactive. During this exercise there was relatively little activity in SCM when latissimus was engaged (7 ± 4%), whereas when latissimus was not engaged this fell to 5 ± 4%.

**Figure 6:**
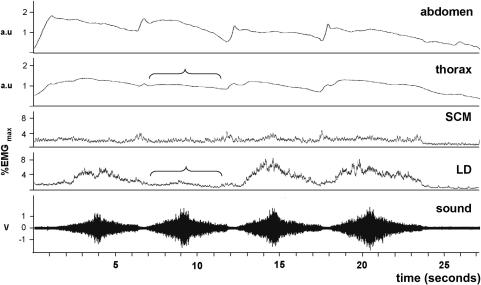
Exercise 5 as sung by mezzo 2 when asked to engage LD. The bracket indicates the second repetition of the exercise when LD was not engaged, resulting in a reduced expansion of the thorax. a.u., Arbitrary units.

Figure 6 shows four repetitions of task 5 by mezzo 2. The subject had been instructed to use LD, however, during the second repetition of the sung phrase, the muscle was not engaged. This resulted in a reduction in amplitude of the plethysmograph trace from the thorax, which failed to reach the levels seen in the phrases sung before and afterward. Taking place within a sequence of activation of the muscle, this strengthens the case for its activity being functionally related to thoracic expansion. It did not, however, cause a noticeable alteration in the amplitude or shape of the signal from the sound channel.

#### Coloratura exercise (task 6)

**Figure 7:**
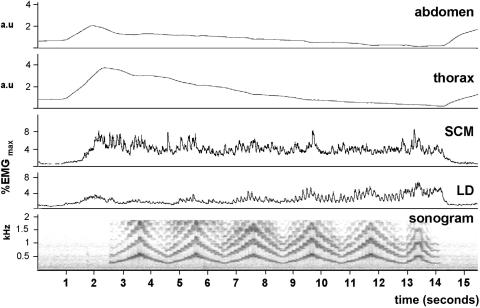
The coloratura exercise, sung by mezzo 1. The sound channel is displayed as a sonogram and the individual notes can be seen to correlate with pulses of activity in LD, whose amplitude increases progressively toward the end of the exhalation. a.u., Arbitrary units.

This exercise is very demanding in terms of breath control and only half the singers were able to complete it fully under a single breath (Figure 7). As a result, it was to be expected that toward the latter part of the breath LD would become increasingly active, and this was indeed the case for all singers. In four of the singers, the raw EMG trace appeared to show bursts of activity in the muscle in phase with the individual notes sung, and this was confirmed by detailed matching of the integrated EMG trace to the sonogram of the sound channel (not shown). The singers chose their own speed for the coloratura exercise. We looked to see whether this was related to vibrato frequency, perhaps because of a preferred resonance frequency for the neuromuscular component of the vocal apparatus of each singer. However, the mean coloratura note frequency was 0.7 Hz faster than the mean vibrato frequency (6.8 Hz compared with 6.1 Hz). SCM showed a variable level of activity during this exercise (it is particularly high in Figure 7), often rising and falling in phase with LD but not increasing in amplitude toward the end of the breath.

#### Aria finale (task 7)

**Figure 8:**
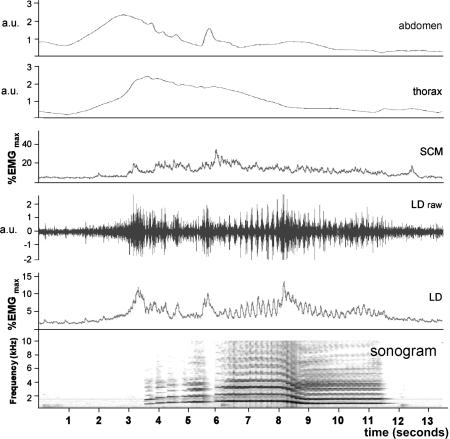
Aria finale exercise from “Una voce poco fa” by Rossini sung by soprano 2. Oscillations in frequency representing vibrato are visible on the sonogram. The integrated EMG trace for LD shows rhythmic activity in phase with this during the two sustained notes at the end of the phrase (6–11.5 seconds into the recording). The raw EMG trace for LD is included to demonstrate that this is free of movement artifact associated with the vibrato. a.u., Arbitrary units.

**Figure 9:**
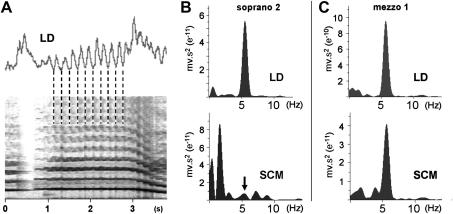
**A.** An enlarged view of a section of Figure 8 (soprano 2) showing the integrated EMG from LD and the sonogram of the first held note of the aria finale exercise. This reveals phase locking between activity in the muscle and the pitch oscillations of the vibrato. **B,C.** Power spectra of the integrated EMG traces for LD and SCM for this note as sung by soprano 2 and mezzo 1. The prominent peaks just above 5 Hz match the vibrato frequency. Both singers have a strong signal for LD. Although mezzo 1 also shows a large peak for SCM, this is very weak for soprano 2 (arrow).

This task involved a sequence of five ascending notes, followed by two sustained notes linked by a glissando (Figure 1), all sung at full volume with the projected voice. In four of the singers (mezzos 2 and 3, sopranos 2 and 3), bursts of activity in LD were associated with each note of the ascending series and coincided with an inward movement of the abdomen (Figure 8). When the sustained notes were sung (during which mean peak activity reached 22 ± 10%), there was a clear evidence of pulsatile activity in the raw EMG trace of the three mezzos and soprano 2. This was even more obvious in the integrated trace (Figure 8). From the sonogram of the audio trace this was seen to be phase locked with the pitch oscillation of the vibrato (Figure 9A), and the power spectrum of the integrated trace revealed a pronounced peak at vibrato frequency. Although pulses of activity were not so obvious in the raw EMG trace for LD for mezzo 3, power analysis of the integrated trace also showed a strong peak at vibrato frequency. The prominence of the EMG oscillation and the magnitude of the associated peak in the power spectrum were less in the second (and lower) of the sustained notes, although still clearly evident. In all singers, there was a significant amount of activity in SCM (17 ± 9%) throughout the exercise, although it varied in magnitude between individuals. In only two cases (mezzos 2 and 3) did the raw EMG trace appear to be obviously pulsatile, but power analysis revealed activity at vibrato frequency in four singers (all three mezzos and weakly in soprano 2—Figure 9B,C). Only for soprano 1 was there no evidence of this in either muscle during the sustained notes, despite a pronounced vibrato being visible on the sonogram.

## Discussion

### Muscle structure

LD is a very broad muscle at its origin from the vertebral column and thoracolumbar fascia, but the area of its insertion onto the humerus is only about 45 × 4 mm. Even some distance from the insertion where it is purely muscular, its cross section is small compared with its broadest regions.^22^ It lacks an inner tendinous scaffold and only approximately the last 7 cm adjacent to the attachment site is tendinous. Most of the fibers are much shorter than the muscle itself, but they are linked by numerous myomyonal junctions that can be end to end or end to side. Small myotendinous junctions also link the fibers in a similar fashion. These impart a net like structure to the muscle.^11^ There are no well-defined zones of innervation, instead the endplates are scattered throughout the muscle and there is good evidence that many fibers are multiply innervated. Campbell^12^ emphasizes that “contraction of the muscle as a whole compresses the lower thorax,” implying that in this situation the attachments to the ribs rotate them inferiorly and so act as insertions. In the present study we see such action at the end of the prolonged blow and prolonged phonation exercises (tasks 1 and 4). The same can be said of the pulses of increasing amplitude in the muscle during the coloratura exercise, presumably contributing to the driving force for the individual notes. The slips running to the ribs should not, therefore, be viewed simply as bundles of fibers linking the thorax to the humerus but rather as attaching the underside of the muscle sheet to the ribs. Their role in maintaining chest circumference could also be viewed in the same light. When the muscle is activated strongly after the initial phase of inhalation, the width of the chest can be felt to increase. If the chest is held high by the external intercostals and scalenes (thus resisting any downward rotation of the ribs), contraction of latissimus “as a whole” would instead tend to flex the ribs outwards. This might explain its observed behavior during inhalation better than the conventional view that the slips attached to the ribs pull them upward toward the humerus.

SCM runs from the mastoid process of the temporal bone, just behind the ear, to the sternum and adjacent region of the clavicle. When active on one side only, it rotates the head to the opposite site and tilts it upwards. When both are active together, they draw the head forward. If this movement is resisted by other muscles, the ribcage is pulled upward, accounting for its role in inhalation. It is known to be recruited during inspiration, usually when lung capacity is increased above 75% vital capacity.^12,23^ Consistent with this, we found it to be active during very deep inhalations, or when a short sharp inhalation was made, for example, between phrases. Its tendinous lower attachments can often be seen to rise in singers, and indeed in speakers before a prolonged period of phonation.

### Activity in LD and SCM during nonsinging tasks

The results from the nonsinging tasks essentially confirm previous long established descriptions of the role of LD and SCM in respiration.^9,10^ Both muscles are active when a deep inspiration takes lung volume close to 100% of vital capacity. At the end of a forced expiration, LD but not SCM becomes increasingly active again. During sharp forced expirations, comparable with the explosive expirations of coughing, we observed that SCM is sometimes active during the brief preceding inhalation, whereas LD shows an intense burst of activity at the start of the expiratory phase. Gray and Williams^9^ and Campbell^12^ state that the activity in LD can be confirmed by palpation, Campbell quoting Beevor^24^ as his authority, although it is a simple enough matter to demonstrate it for oneself! During breathing against resistance, a sustained burst of activity in LD is associated with the increase in chest circumference and is consistent with the pattern reported by Cala et al^16^ and Orozco-Levi et al^15^; this is generally accompanied by matched activity in SCM. It is interesting to note that LD was not engaged during expiration during this task, perhaps because breathing was rather shallow under these circumstances.

In studies of the role of LD during speech,^18,19^ subjects were asked to repeat the syllable “ma” continuously at a frequency of about 2 Hz or to count for as long as possible. The tasks of prolonged blowing and the sustained nonsung vowel in our study made similar respiratory demands on the subjects, and the results were comparable with a progressive increase in activity from LD during the final seconds of the breath. This clearly applies equally to singing because during the coloratura task, which required all of (or sometimes more than) the singers’ total vital capacity for completion, it became strongly active toward the end of the exercise. In this context, it therefore has an expiratory role.

### Activity in LD during projected singing

The trained singers in our study, all used LD during projected singing. They were also able to disengage it on request. The use of LD by singers has in the past been dismissed as a product of clavicular breathing and so deemed undesirable.^3,4^ Clavicular breathing is characterized by Miller^3^ as “raising the clavicles with the intake of breath, and letting them fall in expiration” and he went on to write that during the expiratory phase “the sternum and ribs fall as do the clavicles.” However, the observation does not appear to be founded on any objective evidence but to be based on an incomplete appreciation of the actions of the muscle. In our singers, the shoulders did not rise in a fashion that would be consistent with clavicular breathing, and indeed the muscle was not engaged during inhalation unless in preparation for a breath of maximal duration. It became active immediately after the initial rapid phase of inhalation and was linked to a maintained or increased chest circumference. A considerable level of activity was maintained during the sung phrase, increasing or decreasing slightly with pitch. The chest did not collapse during phonation, and although its circumference did decline somewhat over time, it remained throughout the vocalization at a greater level than when the muscle was not engaged. Chest circumference only reduced markedly when activity in the muscle ceased and phonation ended almost simultaneously. The muscle, therefore, appears to be used to maintain an expanded or partially elevated chest, sometimes described by singers as the noble^25^ or pear-shaped down^26^ posture.

There is still some debate as to why an elevated chest posture might be advantageous.^26^ One suggestion is that the high chest is linked to a lower laryngeal position^27,28^ that is favorable for vocal projection. It has also been proposed^29,30^ that keeping the chest high initially and using the abdominal muscles as the main driving force even at high lung volumes may have several other advantages. First, the firm platform formed by the upwardly displaced diaphragm and tense abdominal wall means that any contraction of expiratory muscles of the thorax, which are kept stretched by this procedure, is more efficiently translated into changes in subglottic pressure rather than into deformations of the chest or indeed the abdominal walls. In this state, the muscle fibers of the diaphragm are also stretched, which is advantageous if it must be used for small rapid intakes of breath at relatively high lung capacities.^30^ Second, the rib cage contact area with the lungs is about three times that of the diaphragmatic surface, through which the abdominal muscles must act. Thus small movements of muscles of the chest wall are an effective means of exerting the fine control over baseline subglottic pressure that is so necessary for singing. This is to some extent consistent with the influential notion of “fixed diaphragmatic breathing” as promoted by the English school of singing pedagogy. Miller^3^ reports that in this technique, singers were instructed to raise the chest and expand the ribs laterally at the same time as the abdominal wall is drawn inward. Slater,^31^ a major advocate of this technique, goes on to state that allowing distension of the abdomen during inhalation makes it “impossible to obtain a free action of the ribs and a proper expansion of the chest.” However, unless the abdominal muscles are allowed to relax during inhalation, the amount of air taken in will be significantly limited and today many singing teachers specifically emphasize the importance of this action.^7^

### Activity of SCM during singing

The behavior of SCM during singing has received a considerable amount of attention recently by Pettersen and Westgaard.^1,32,33^ These authors propose that it produces “a counterforce to expiratory movement to prevent too rapid a reduction of upper thorax circumference and hence too high a subglottic pressure”—in other words, it contributes to respiratory braking, which is used to resist the elastic recoil forces of the chest wall at high vital capacities. They also note that during glissandos, its activity increases with rising pitch. The vocal range required by the exercises our singers carried out never extended over more than about an octave, and so this was only barely detectable in a few of their traces. Pettersen’s subjects sang whole arias in many cases, and he found that activity levels of SCM during singing were higher than those seen during inhalation, and greatest when the singing task was most demanding. The same was certainly true for the aria finale phrase that our subjects performed.

In discussing the role of SCM in very demanding singing tasks such as this, Pettersen uses a slightly different expression to the one quoted earlier, stating that the counterforce it produces provides “better control over the expiratory movement.”^7,33^ The meaning of this is not absolutely clear, but presumably the muscle helps maintain a high chest posture so that the forces generated by the expiratory muscles act from the bottom up. Of course, another possibility is that it plays no positive role in singing phonation and that its activity is a manifestation of neck tension. Some singing teachers suggest that it contributes to clavicular breathing,^34^ but as it inserts onto the sternum and the immediately adjacent part of the clavicle, this seems unlikely. Pettersen explored the question using biofeedback to determine whether singers can reduce activity levels in accessory respiratory muscles while maintaining performance. In investigations involving SCM and the upper part of trapezius (a muscle which does raise the shoulder and often exhibits stress-related tension), biofeedback led to a marked reduction in activity in the latter. For SCM, on the other hand, there was only a small reduction in the level of activity during phonation (but not inhalation) and no change in the pattern of its activation. It was concluded that although trapezius was apparently being overused by the singers, the evidence was less clear for SCM, making it more likely that it plays a positive role in singing.

### Muscle activity associated with vibrato

In classical singing, vibrato is predominantly a fluctuation in sound frequency, typically of around 50 cents.^35,36^ In popular music, a vibrato based on fluctuations in sound intensity is sometimes present and is likely to be because of oscillations in subglottic pressure.^37^ This probably reflects differences in training. Classical singers are taught to keep the position of the larynx as stable as possible, whereas singers of popular music may not resist rapid fluctuations in laryngeal position during vibrato to the same extent. In classical vibrato, fluctuations in intensity are sometimes superimposed on those of frequency.^38^ This could be seen in some of the sound traces we recorded and is also visible in many other reports on classical singers.^39^ Although the rate of vibrato varies somewhat between individuals, measurements by Seashore^40^ from 29 singers found values of 5.9–7.8 Hz (mean 6.6 Hz) with no systematic gender differences. Prame^35^ analyzed 10 singers (seven females, three males) and reported a range of 5.5–6.7 Hz (mean 6.0). The vibrato frequencies of our singers ranged from 5.2 to 7.0 Hz (mean 6.1). It has been demonstrated using electromyography that this is associated with synchronized rhythmic activity in some laryngeal muscles, primarily the cricothyroid,^37^ although the lateral cricoarytenoid and thryoarytenoid may also contribute.^39^

It is clear from our results that some singers generate activity in respiratory muscles that is phase locked with the vibrato. The paucity of studies on the possible involvement of respiratory muscles in vibrato has led to considerable prominence being given to a small number of weak sources. Vennard^4^ proposed that “tremor” in respiratory muscles may be a contributing factor on the basis that this can be felt on the “epigastrium” of some singers. In support, he quotes what are apparently unpublished studies by Osborne and Wade to suggest that this does not apply to the diaphragm. An electromyographic study by Appleman and Smith^41^ reported that the internal and external obliques, rectus abdominus, and LD are not active in synchrony with vibrato. However, all but one of our subjects exhibited activity at vibrato frequency in LD and four of the six in SCM. Although there have recently been a considerable number of electromyograph studies of a range of respiratory muscles during singing (see review by Pettersen^1^), their activity was described over timescales typical of the gross movements of the thoracic wall and so did not reveal whether patterns of activity at vibrato frequency were present.

During the production of vibrato, many structures both muscular and nonmuscular vibrate. The high band pass filtering of the electromyograph signal in our study eliminated any movement artifact at vibrato frequency from recordings, but the movement may nevertheless help to synchronize rhythmic activity in respiratory and laryngeal muscles. Nonpathological muscle tremor and vibrato are thought to be controlled both by oscillatory circuitry within the central nervous system and by peripheral feedback.^42,43^ Under steady state muscle contraction, muscle spindle afferent output, for example because of passive rhythmic stretching, can lead to coordinated motor unit activity. This is typically in the 6–10 Hz range, probably because of the delays inherent in spinal reflex pathways, although it can be reduced further by increasing the inertia of the moving structures.^44^ Such a mechanism could, therefore, reinforce and phase lock rhythmic muscle activity at vibrato frequencies.

The notion that respiratory muscles may contribute to vibrato is not without precedent. The respiratory demands of flute playing share a number of features with singing. Pressure generated in the airway is low, flow rates are high and respiratory braking is important when lung capacity is high. Flautists typically use a marked vibrato and many believe that this is controlled by the muscles of the abdominal wall or the diaphragm. However, direct examination of the larynx during playing has revealed that the movements of the vocal folds often play an important role,^45,46^ but in some (although not all) flautists, the diaphragm and other inspiratory muscles provide rhythmic respiratory braking at vibrato frequency at the beginning of the breath, whereas similar patterns of activity in the abdominal muscles maintain the vibrato thereafter.^47^ In the present study, the rhythmic activity was clearly visible in some raw or integrated EMG traces, however, in other cases, it only emerged from spectral analysis of the integrated signal. This of course raises the question of how significant the rhythmic activity in the muscles is for the production of the vibrato. Presumably, it causes fluctuations in subglottic pressure, and although this will make a small contribution to pitch oscillation, it may be more significant for the fluctuations in sound intensity that are a variable feature of vibrato on classical singers. It should be noted, however, that we also saw marked fluctuations in intensity in singers who did not show rhythmic activity in LD and in it is possible that in these individuals, other respiratory muscles may be involved.

### Variation between singers

The singers we studied clearly did not all use LD and SCM in the same way (eg, when generating vibrato) and such variability is a common feature of objective studies of vocal technique. As most investigations have access to only limited numbers of subjects, one should never be too dogmatic when drawing conclusions about how particular effects are produced. Indeed, a recognition of this variability is important for vocal pedagogy. It is striking that individual trained singers are generally very consistent in their behavior even when tested on the same tasks over considerable time periods.^48–50^ This suggests that different singers may achieve the same ends in one of several possible ways. Although a number of possible reasons have been put forward, the question has not been studied systematically despite its importance for the training of singers. Even within the classical tradition, there are many different schools of thought on technique, although as discussed by Miller^3^ these may have rather different objectives. In addition, the demands made by different repertoires vary considerably. The level of experience of the subjects is obviously one significant factor. For example, professional opera singers tend to show greater activity in a range of respiratory muscles when compared with students,^1^ although excessive respiratory muscle activity may also be used to compensate for a lack of proficiency in vocal projection. Gender, age, and voice type may also be significant sources of variability. A question that is rarely discussed in this context is the possible significance of body morphology. Based on a study of just 12 individuals, Hoit and Hixon^51^ proposed that speech breathing in endomorphs is driven predominantly by abdominal movement, that of ectomorphs more by chest movement, whereas mesomorphs use an intermediate strategy. Although a subsequent study involving considerably more subjects divided into six morphological categories failed to confirm this observation,^52^ a number of methodological problems have been raised with it,^53^ and so the question remains open. Furthermore, it has yet to be investigated in the more demanding context of projected singing. This question could not be addressed in our study because of the small degree of variability among our subjects who although ranging in height from 160 to 176 cm, all had body mass indices (BMIs) of 21–22. Their plethysmograph traces showed similar degrees of expansion of the chest and abdomen during breathing. By contrast, recordings of the same set of singing tasks in a tenor who was 192 cm tall and had a BMI of 35 revealed a pattern that was heavily dominated by abdominal movement, which would be consistent with the predictions of Hoit and Hixon.^51^

## Conclusions

Some singing teachers believe that activity in LD and SCM is indicative of clavicular breathing, but there was no evidence for this in the singers who took part in our study although both muscles were active during inhalation at lung volumes close to 100% vital capacity. Instead, LD was consistently active during projected singing and appeared to contribute to keeping the chest expanded during exhalations that only partially deflated the lungs. However, when lung volume fell closer to residual volume, it took on a rather different role; that of expelling air from the lungs. This apparently paradoxical behavior is consistent with previous studies of the muscle in speech and other nonsinging tasks. The consequences of its contraction appear to depend on the amount of freedom the ribs have to move because of the action of other respiratory muscles. In some but not all singers, peaks of activity in LD were phase locked with individual notes during coloratura singing and these became increasingly prominent as lung volume fell. This may contribute to small increases in subglottal pressure at the initiation of each note. Fluctuations in the activity of the muscle was also sometimes synchronized with vibrato. The fact that all of the singers did not use the muscle in these ways emphasizes the importance of acknowledging and understanding why the same ends may be achieved by different means in experienced performers. In contrast to LD, levels of activity in SCM were generally much lower during projected singing, and it was not recruited increasingly as lung capacity fell. This is consistent with its anatomy.
